# Wear Enhancement of Wheel-Rail Interaction by Ultrasonic Nanocrystalline Surface Modification Technique

**DOI:** 10.3390/ma10020188

**Published:** 2017-02-16

**Authors:** Seky Chang, Young-Sik Pyun, Auezhan Amanov

**Affiliations:** 1 Korea Railroad Research Institute, Uiwang 16105, Korea; seky@krri.re.kr; 2 Department of Mechanical Engineering, Sun Moon University, Asan 31456, Korea; pyoun@sunmoon.ac.kr

**Keywords:** 60 kgK rail material, hardness, compressive residual stress, wear resistance, UNSM 5

## Abstract

In this study, an ultrasonic nanocrystalline surface modification (UNSM) technique was applied to normal and heat-treated rails made of 60 kgK steel to enhance the wear resistance of the wheel-rail interaction. The hardness and compressive residual stress values of the untreated and UNSM-treated rails were measured by the Brinell hardness tester and X-ray diffraction technique, respectively. It was found, according to the measurement results, that the hardness was increased by about 20% and 8%, whereas the compressive residual stress was induced by about 52% and 62% for the UNSM-treated normal and heat-treated rails, respectively. The UNSM-treated normal rail showed a slightly higher hardness than the heat-treated rail. The wear resistance of rails with respect to rotating speed and rolling time was assessed using a rolling contact wear (RCW) tester under dry conditions. The RCW test results revealed that the wear of the UNSM-treated rails was enhanced in comparison with those of the untreated rails. Also, the wear amount of the rails was increased with increasing the rotation speed. The UNSM-treated normal rail exhibited the highest wear resistance with respect to the rotation speed. The wear mechanisms of the rails are also discussed based on microscopic images of the worn out surfaces.

## 1. Introduction

A key part of reducing railroad costs and improving safety is through better management of wheel and rail profiles to extend life, reduce vehicle and track maintenance, and improve vehicle stability. The increased energy dissipated at the wheel-rail interaction from wheel/rail profile mismatch and the friction/wear directly affects the fuel required to haul a train. Twenty fields of wheel-rail interface research, among more than thousands of studies, were sorted and discussed concisely [1]. The wear and fatigue life of wheel-rail interactions depend greatly on properties such as contact stress, surface hardness, and compressive residual stress, which in turn are controlled by the wheel-rail microstructure and the way they interact [2]. This interaction also affects fuel consumption, derailment risk, and vehicle stability [3]. Most of the damage on the rail is caused by rolling contact wear (RCW) and rolling contact fatigue (RCF) since train wheels and rails are always in contact with dynamic motion in addition to environmental influences. Once the rail surface is damaged, the wheel-rail profile is mismatched and wear is accelerated by friction [4]. The rail surface condition becomes worse to cause failure at the weak points. Before the micro-cracks initiated at the weak points grow over the designed threshold criteria, the rail surface should be inspected and repaired.

There have been many studies on rail damage and maintenance in order to secure safety on the railway [5,6]. The wear resistance of the rail-wheel interface was studied earlier using a tin disk testing method, which is a simple way to evaluate the wear characteristics of materials [7]. It has been also reported earlier that the wear rate of the rail-wheel interface can be reduced with an increase in hardness [8]. In addition to the wear behavior, the fatigue strength of rail materials has been studied by many scholars. For example, Brunel et al. investigated the fatigue behavior of railway steels including steel grade and sliding condition effects by using a rolling contact fatigue testing machine [9]. They concluded that the classification of the material grades and sliding conditions can be selected by the RCF test method. Moreover, surface and heat treatment methods can improve the wear and fatigue behaviors of the rail-wheel interface. The wear behavior of bainitic rail-wheel steels was investigated using a wear tester under reciprocating dry sliding conditions by Sharma et al. [10]. They found that the bainitic steels exhibited a better wear resistance compared to the existing pearlitic and ferritic-pearlitic steels due to the presence of a typical bainitic morphology. Also, the influence of laser dispersed treatment (LDT) on RCW and RCF behaviors of railway wheel steel was investigated by Zeng et al. [11]. It was found that the LDT effectively improved both the wear resistance and RCF behavior of the railway wheel steel due to the formed fine martensite and retained austenite.

The surface of the rail head is open to foreign objects, vibration, slip, friction and stress. Thus, the rail surface is hardened by heat treatment at the critical sites. The heat treatment of the rail head is often applied to strengthen the surface of the rail in use under severe conditions, but there is still ongoing demand to increase the wear resistance and rolling contact fatigue strength. Moreover, the friction and wear behavior is central to understanding and optimizing the wheel-rail interaction due to the vehicle dynamics and traction at braking [12]. Also, deformation generated under contact stress may lead to surface and sub-surface shear stresses that increase rolling resistance and, more importantly, contribute to decreased wear resistance and deteriorated fatigue [13]. No ideal material that does not wear and or suffer from fatigue for railway and wheel applications has been found yet.

Therefore, there is a need to increase the mechanical properties of both rails and wheels in order to improve the wear and to extend the fatigue life. Molyneux-Berry et al. investigated the effects of wheel-rail contact conditions on the microstructure and hardness of railway wheels [14]. They concluded that the microstructure and hardness have a significant influence on the wheel-rail contact conditions. Wang et al. also reported on the friction, wear and surface damage behavior of rails and wheels with different hardnesses [15]. It was found that the hardness of the wheel had no effect on the rolling friction coefficient of the wheel-rail interaction, but the wear volume decreased with increasing the hardness of the wheel. Hence, it can be concluded that the surface hardness and microstructure play an important role in determining the friction, wear and surface damage of rail-wheel interactions. Hence, the objective of this study is to improve the wear and to extend the fatigue life of wheel-rail interactions and to investigate the possibility of replacing the heat treatment process with a UNSM treatment process. The present study was performed to investigate the effect of surface layer modifications on hardness, the compressive residual stress, and the wear and rolling contact fatigue performances of two types of rails: one was a normal rail and the other one was a heat-treated rail.

## 2. Materials and Methods

### 2.1. Specimen Preparation

In this study, a couple of normal and heat-treated rails made of 60 kgK material were provided by a Korean rail manufacturing company [16]. The rail was heat treated at a temperature of 800 °C by induction heating and quenched in water to get sorbate from austenite structure, which resulted in increase of hardness and wear resistance. The chemical composition of 60 kgK rail steel is listed in Table 1. The 60 kgK rail is usually used for high speed train and high passing tonnage railways like metro and main intercity lines. This rail was designed to satisfy the local standard and weights 60 kg per meter. The specimens with dimensions of 25 mm in diameter and 4 mm in thickness were cut from those rails for the wear tests. Prior to the UNSM treatment and wear tests, the specimens were polished by SiC sandpapers in order to keep the identical surface roughness for each specimen. The specimens were cleaned with a mixture of ethanol and deionized water for 10 min using an ultrasonic bath to remove the particles and impurities from the surface of the specimens.

### 2.2. UNSM Treatment Process

**Figure 1:**
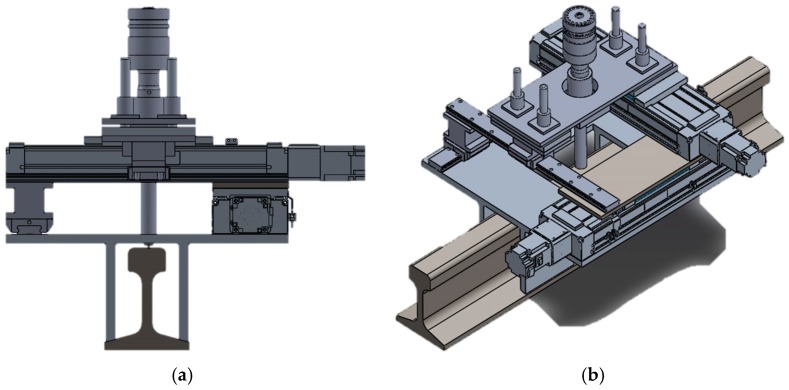
An assembly of the newly designed portable UNSM treatment device for railway application. It has an electronic system that can coordinate the treatment area by two electronic motors which moves along the LN guide. The impact load of the treatment can be controlled by radially applied dead load. This portable device can be brought to the field and applied to the rail. Two different views are shown as: (**a**) Side view; (**b**) 3D view of a portable UNSM treatment device.

The UNSM technique has received much attention due to its versatility in improving several properties such as mechanical, tribological and fatigue of metallic materials by the application of severe plastic deformation (SPD) at the surface and sub-surface by striking the specimen surface up to 20,000 times per second with the hard balls made of tungsten carbide (WC) ball. These strikes can be considered as a micro-cold-forging. In this UNSM treatment process, not only the static load (*P_st_*), but also the dynamic load (*P_dy_ = P_st_* sin 2π ft) is exerted to the surface of the specimen, where *P_st_* is the static load, *f* is the frequency and *t* is the time. Some of the specimens were polished, while the others were treated by a UNSM treatment process under the optimized treatment parameters as listed in Table 2. The optimum treatment parameters were determined according to the surface quality and hardness measurement results. These parameters allow to control the mechanical properties and the thickness of plastically deformed layers along with grain size. A set-up of the newly designed UNSM device can be seen in Figure 1. Such mechanical contact produces severe plastic and elastic deformation in the metal surface layer, which induces deep compressive residual stress and refines the grain size to increase the surface hardness. The UNSM technique is effective in increasing surface hardness, compressive residual stress and refining grain size and thus increase wear resistance and rolling contact fatigue strength. So the UNSM can be applied not only to strengthen new rail, but also to restore the damaged rail to equal or even better in wear and rolling contact performance. More details of the UNSM treatment process can be found in our previous studies [17,18,19,20].

### 2.3. Rolling Contact Wear and Rolling Contact Fatigue Tests

Rail wear is affected by several factors such as rail material property, track layout and geometry, curve diameter, axle loads in addition to train speed and maintenance, etc. [21]. The friction and wear behavior of the specimens was assessed according to ASTM G99 standard using a ball-on-disk tester under dry conditions. The wear resistance of the rails with respect to rotating speed and rolling time was assessed using a rolling contact wear (RCW) tester under the conditions as listed in Table 3. The rolling contact fatigue (RCF) tests were performed on the normal and UNSM-treated normal specimens with a counter surface of Si_3_N_4_ ceramic ball with a diameter of 7.14 mm in dry conditions as listed in Table 4. The fatigue limit of the specimens was determined by a vibration sensor where it halts the machine when the vibration value reaches a certain value.

### 2.4. Specimen Analysis and Measurement Details

The changes in the surface properties were measured to estimate the effect of UNSM with respect to surface hardness and compressive residual stress in the surface layer. The hardness and compressive residual stress values of the untreated and UNSM-treated rails were measured by the Brinell hardness tester (Mitutoyo MVK-E3, Mitutoyo, Chiba, Japan) at a load of 300 gf with a dwell time of 10 s and non-destructive X-ray diffraction technique (Proto iXRD-portable, Oldcastle, ON, Canada) respectively. The surface roughness and wear profiles were obtained using a two-dimensional (2D) surface profilometer (Mitutoyo SJ-210, Mitutoyo). The wear rate of the specimens after wear tests was quantified based on the wear track profiles. The worn out and damaged surfaces were characterized using a scanning electron microscopy (SEM, SNE-3000M, SEC Inc., Suwon, Korea) and an energy dispersive X-ray spectroscopy (EDX, Quantax XFlash 6, Bruker, Billerica, MA, USA).

## 3. Results

### 3.1. Hardness and Compressive Residual Stress

**Figure 2:**
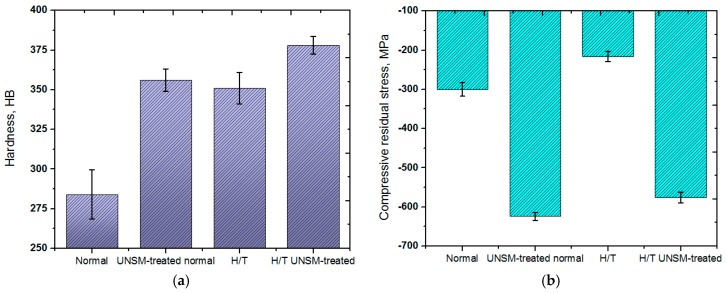
Comparison in hardness (**a**) and compressive residual stress (**b**) of the normal and heat-treated rails before and after UNSM treatment process.

Figure 2a shows the comparison in surface hardness for the normal and heat-treated specimens before and after the UNSM treatment process. It can be seen that the UNSM treatment process increased the hardness from 284 to 356 HB, and 351 to 378 HB for the both the normal and heat-treated specimens, respectively. As is obvious, the increase in surface hardness was more noticeable with the normal rail in comparison to the heat-treated rail. The hardness of the heat-treated rail was a bit higher even before the UNSM treatment process compared to that of the UNSM-treated normal rail. The increase in hardness after the UNSM treatment may be explained following the Hall-Petch relationship, where the hardness of a material depends on the grain size. After the UNSM treatment, the coarse grains of the material were refined into nano-scale grains [22]. A review on the effect of rail hardness on wheel-rail interactions was reported earlier by Lewis et al. [23], where it was shown that it has a significant effect on the performance of wheel-rail interactions. A comparison in compressive residual stress of the normal and heat-treated specimens before and after the UNSM treatment process is shown in Figure 2b. It can be also seen that the compressive residual stress was induced up to −624 and −576 MPa for the normal and heat-treated specimens after the UNSM treatment process, respectively. The surface compressive residual stress measured before the UNSM treatment was probably affected by the cutting and machining process of the specimens since, in general, the surface layer is deformed by contact with cutting tools. It is obvious that the UNSM treatment process successfully induced a compressive residual stress in the surface layer. It is well known that the possible reasons for increasing compressive residual stress are the reduction of the grain size [24] and the increase of the lattice distortion [25] caused by residual stresses or dislocations. Grain boundaries, dislocations, and other atomic-level microstructural defects have been experimentally proven to effectively accelerate the diffusion of interstitial atoms. As a result, the increased hardness and induced compressive residual stress after the UNSM treatment process may be attributed to a kind of hardening effect by a WC ball tip hitting the rail surface through the ultrasonic system at a high frequency of 20 kHz and to the refinement of coarse grains into nano-sized grains. The increase in the hardness of the normal rail was due to the compressive residual stress developed in the surface layer by the UNSM treatment. However, in the case of the heat-treated rail, the metal texture was changed by the heat treatment, which contributed to increasing the surface hardness compared to the normal rail. It was reported earlier that nanocrystallization and grain size refinement by the UNSM treatment process are responsible for the increase in the mechanical properties including compressive residual stress [26,27]. It was reported in our previous study on wear and chattering characteristics of rail materials that the presence of a modified top surface layer along with a plastically deformed layer of normal and heat-treated specimens by UNSM treatment with a thickness of about 80 and 40 µm was observed [20]. The mechanical properties of layers formed by severe plastic deformation (SPD), in which a very large plastic strain is imposed on a bulk process in order to refine the coarse grains into nano-sized grains, tend to be higher than those of non-deformed layers. Moreover, the mechanism of grain size refinement after the UNSM treatment can be explained in the following procedures: (i) dislocation accumulation; (ii) the formation of subgrain boundaries; (iii) some dislocations are annihilated at subgrain boundaries to increase the misorientation angels; (iv) finally, balance is established between the generation of dislocations by SPD and the absorption of dislocations at grain boundaries [28].

### 3.2. RCW and RCF properties

**Figure 3:**
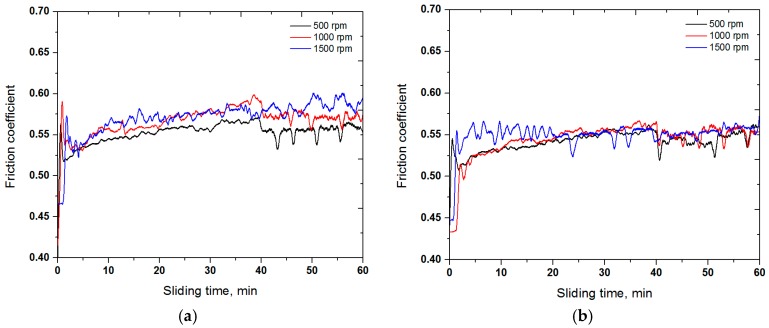

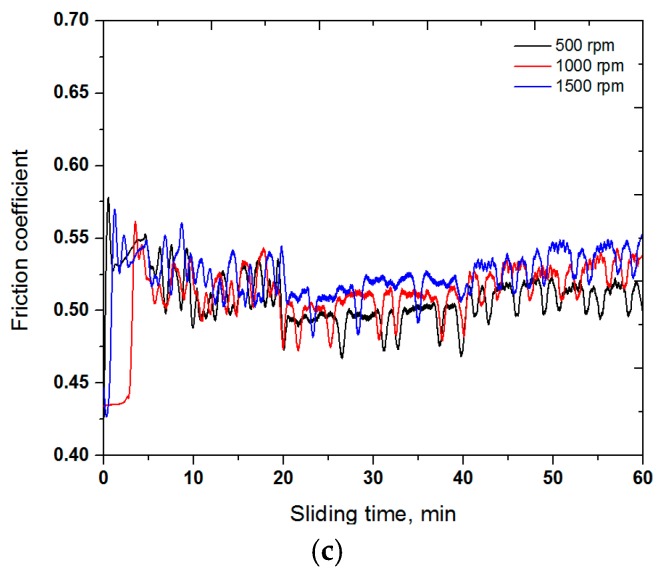
Comparison in friction coefficient of the normal (**a**) heat-treated and (**b**) UNSM-treated normal (**c**) specimens with respect to rotating speed.

Figure 3 shows the friction behavior of the normal, heat-treated and UNSM-treated normal specimens with respect to the rotating speed. It was found that the friction coefficient of the UNSM-treated normal specimens was lower in comparison with the normal and heat-treated specimens. The influence of the rotating speed on the friction coefficient was similar for all the specimens, where the friction coefficient was increased with increasing the rotating speed from 500 to 1500 rpm. This behavior may be associated with the formation of wear particles at the contact interface at higher speeds being greater, which can generate a vibration during rolling, and also with the complete removal of the interfacial layer (wear debris and their oxides) formed during the wear process [29]. The actual contact interface depends on the number of factors such as the size and shape of the nominal contact area, contact pressure distribution, real contact area, etc. [30]. Moreover, the lower friction coefficient of the UNSM-treated normal specimens compared to the normal and heat-treated specimens may be attributed to the increase in hardness and the reduction in surface roughness [18].

**Figure 4:**
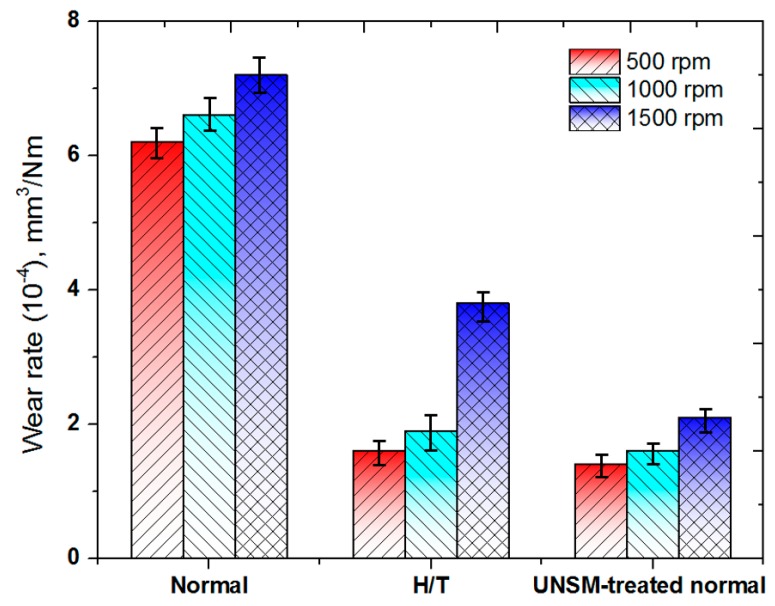
Comparison in wear rate of the normal, heat-treated and UNSM-treated normal specimens with respect to rotating speed.

Figure 4 shows the comparison in the wear rate of the normal, heat-treated and UNSM-treated normal specimens with respect to the rotating speed. The wear rate was increased with increasing the rotating speed for the all specimens. The wear resistance of the UNSM-treated normal rail was the best in the range of all the rotating speeds used for the test. The difference in the wear rate was increased with increasing the test rotating speed between the heat-treated and the UNSM-treated normal rails, where the wear rate of the heat-treated specimen at a speed of 1500 rpm was found to be the greatest compared to the wear rate of the UNSM-treated specimen obtained at a speed of 1500 rpm. Ding et al. investigated the effects of the rotating speed on the rolling wear and damage of the wheel/rail materials [31]. They reported that rolling wear loss was increased for the wheel, whereas it was decreased for the rail specimen. The obtained results are in good agreement with the results shown in Figure 4, where the wear rate was increased with increasing the rotating speed. In addition, in the wear debris generated from the tests with respect to the rotating speed, the shape of the wear debris (not shown here) was in a plate-like form with sizes of about 20–25, 15–20 and 10–15 µm in length for normal, heat-treated and UNSM-treated normal specimens. The size of the plate-like debris decreased with increasing the rotational speed for the all specimens. The UNSM-treated normal rail showed a slightly higher surface hardness than that of the heat-treated rail, as shown in Figure 2a. The compressive residual stress in the surface layer was greater for the UNSM-treated normal rail than in the heat-treated rail, even if the differences in the surface hardness of both rail specimens were not great. It was likely to cause the difference in the wear rate with the rotating test speed. The results revealed that the wear resistance of the UNSM-treated specimens was enhanced due to the increased hardness and the induced compressive residual stress at the top surface layer.

**Figure 5:**
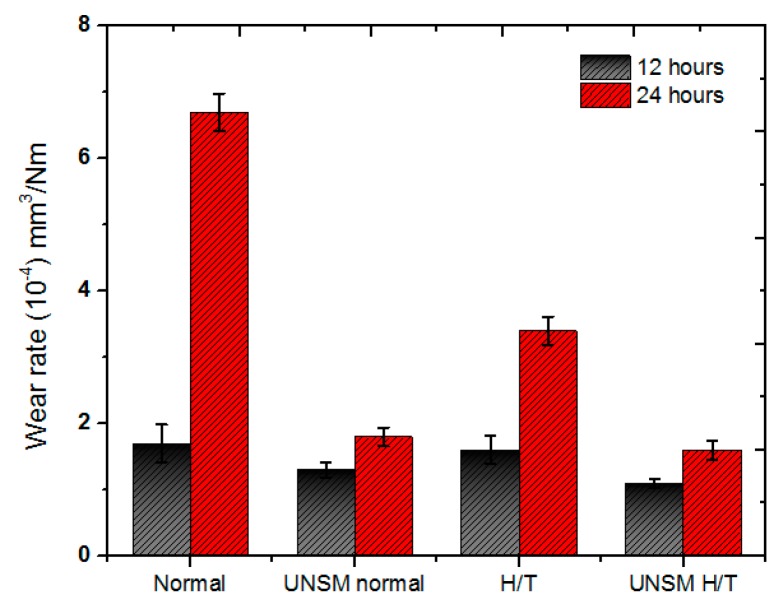
Comparison in wear rate of the normal, UNSM-treated normal, heat-treated and UNSM-treated heat-treated specimens with respect to testing time.

As shown in Figure 5, the wear tests were also performed with respect to testing time. The UNSM-treated normal rail also showed a better wear resistance than the heat-treated rail in the testing times of 12 and 24 h. However, the UNSM-treated heat-treated rail showed the best wear resistance as shown in Figure 5. The wear amount of the normal rail was greatly increased with increasing the testing time from 12 to 24 h compared to the cases of the heat-treated or UNSM-treated rails. Both heat treatment and UNSM treatment were found to be beneficial in protecting the rail from wear, even if the UNSM treatment contributed to the wear resistance of the rail more than the heat treatment in the test conditions.

RCF failure is one of the defects that are often observed on the rail and it is not caused by metallurgical defects, manufacturing faults or wrong treatment of the train operation. It originates from the weak points or fatigue cracks on or near the rail surface [32]. Such defects appear in the shape of the gauge corner crack, squat and shell. The rails are in frequent contact with train wheels which results in a great deal of stress and deformation on the rail. The physical process to determine RCF growth is crack initiation and propagation. The RCF can lead to crack initiation in the rail and finally causes very dangerous catastrophic failure if the defect is not inspected at the right time.

**Figure 6:**
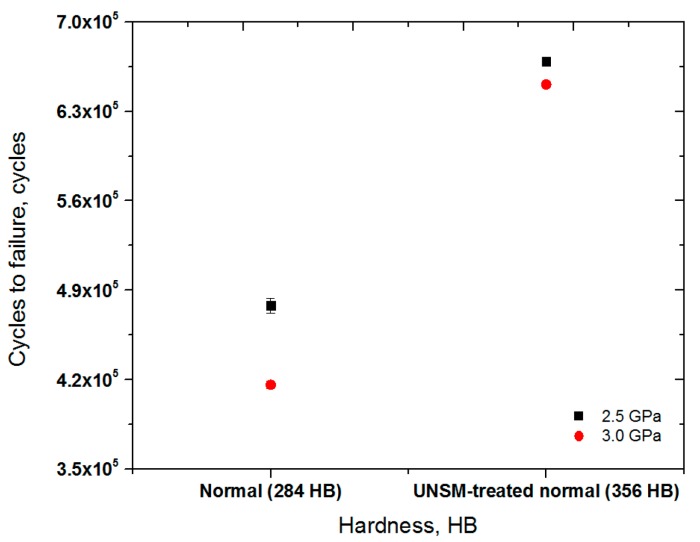
Comparison in cycles to failure vs. hardness of the normal and UNSM-treated normal specimens at two different contact stresses.

It was found based on the RCF test conditions listed Table 4 that the normal rail specimen fails at 478,000 cycles at a contact pressure of 2.5 GPa. The lifetime of the UNSM-treated normal rail was extended by 40.0% with 669,000 cycles to failure at a contact pressure of 2.5 GPa. The fatigue life of the normal rail was 416,000 cycles at a contact pressure of 3.0 GPa and it increased to 651,000 cycles after UNSM treatment at a contact pressure of 3.0 GPa. As a result, the fatigue lifetime of the normal rail specimen was extended by about 57% after the UNSM treatment. The fatigue lifetime of the normal rail specimen was decreased by about 13%, from 478,000 to 416,000 cycles, with increasing the contact pressure from 2.5 to 3.0 GPa, while the fatigue lifetime of the UNSM-treated normal rail specimen was decreased by 2.7%, from 669,000 to 651,000 cycles, for the test time of 24 h. As summarized in Table 5, the UNSM technique effectively reduced the development of RCF at a relatively higher contact pressure in the rails. Figure 6 shows the cycles to failure with respect to the hardness of the normal and UNSM-treated normal specimens at contact pressures of 2.5 and 3.0 GPa. One can see that the cycles to failure of the UNSM-treated normal specimens at both contact pressures were found to be longer than those of the normal specimens. In addition, the cycles to failure of the specimens reduced with increasing the contact pressure, as shown in Figure 6. It is common knowledge that one of the most effective methods of extending the fatigue lifetime is to induce compressive residual stress on the surface of the material [33,34]. Hence, the highly induced compressive residual stress by the UNSM treatment process was mainly responsible for the extension of the fatigue lifetime for the UNSM-treated rail specimens.

**Figure 7:**
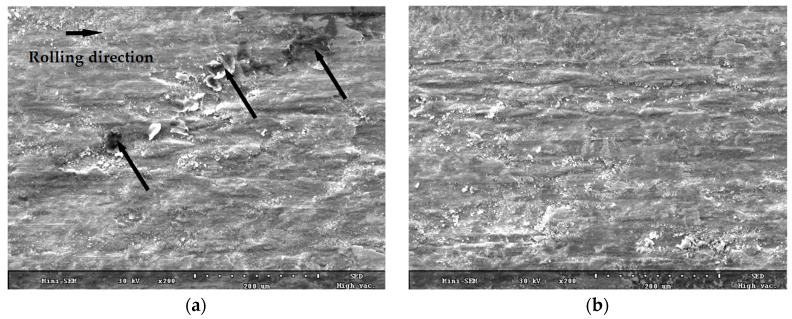
SEM micrographs of (**a**) the normal and (**b**) UNSM-treated normal specimens that failed at 478,000 and 669,000 cycles at a contact pressure of 2.5 GPa, respectively.

**Figure 8:**
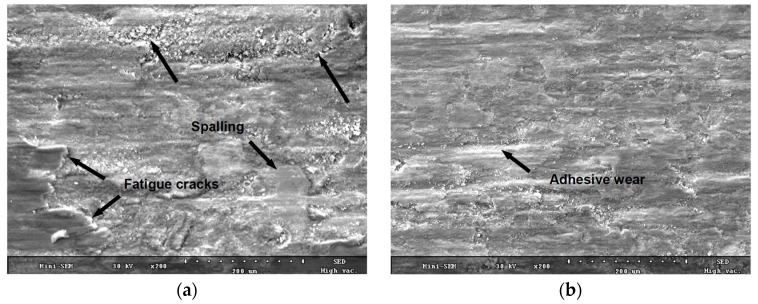
SEM micrographs of (**a**) the normal and (**b**) UNSM-treated normal specimens that failed at 416,000 and 651,000 cycles at a contact pressure of 3.0 GPa, respectively.

Figure 7 shows the SEM images of the fractured surfaces of the normal and normal UNSM-treated specimens which failed at 478,000 and 669,000 cycles at a contact pressure of 2.5 GPa, respectively. Obviously, the surface damage morphology of the normal specimen differs from that of the normal UNSM-treated specimen. It is obvious that the damage characteristic of the specimens is closely related to the wear regimes. In addition, it can be seen that the fracture of the normal specimen is significant compared to the UNSM-treated normal specimen, whereas the surface damage is considerably significant as well and the oxidation wear dominates. As indicated by the arrows in Figure 7a, some huge wear debris/particles and oxidative layers are visible on the fractured surface of the normal specimen, where neither wear debris/particles nor an oxidative layer were observed on the fractured surface of the UNSM-treated normal specimen, as shown in Figure 7b. The chemical state of the worn surfaces was investigated by EDX (not shown here), where the oxidation wear was dominant for the normal specimen at 2.5 GPa. However, no oxidation was found for the UNSM-treated normal specimen at 2.5 GPa, or the normal and UNSM-treated normal specimens at 3.0 GPa, where a Fe element peak was found to be dominant. By increasing the contact pressure, some cracks and delamination can be observed on the surface of the normal specimen, as shown by the arrows in Figure 8. Moreover, the damaged surface became rougher with increasing the contact pressure with obvious peeling damage. The surface of the normal specimen was dominated by the combination of fatigue cracks and adhesive wear, as shown in Figure 8a. The wear mechanism for the UNSM-treated specimens was found to be adhesive wear, as shown in Figure 8b. As a result of fatigue, those cracked zones led to serious spalling of damaged specimens and usually there was visible fatigue cracking in the sub-surface. As the wear became severe at a high contact pressure, the fatigue damage transformed from slight damage to oxidation to spalling and fatigue cracks [11], where the cracks caused by RCF have their origins beneath the surface and may be attributed to the superposition of the maximum sub-surface shear stress over local defects and damages. The induced high compressive residual stress by the UNSM treatment process in the surface layer may withstand the sub-surface shear stress, resulting in extending the fatigue life of rails. The influence of the increased hardness and induced compressive residual stress on the damage at the wheel-rail interaction can be diminished by the UNSM treatment process.

The rail surface was hardened by UNSM as confirmed by the hardness, the wear and the RCF tests. However, when the hardened rail is in contact with the normal wheel, it is expected that the wear rate of the wheel can be increased. In general, it is a well-established fact that increasing the wear resistance of a material on one side of the wheel-rail interaction interface will result in a decrease in the wear resistance on the other side of the wheel-rail interaction [35]. Moreover, it was reported earlier that increasing the rail steel grade did not change the wear resistance of wheel [36]. The wear resistance of the rail increased with increasing the rail grade, but the wear of the wheel was not influenced. Therefore, the application of the UNSM treatment process to both sides of the wheel-rail interaction needs to be considered as a future study direction in order to shed light on the improvement or deterioration of friction, wear and fatigue properties of the wheel-rail interaction. In addition, an understanding in the wear mechanisms and fatigue failure modes of the specimens is needed to emphasize the role of the UNSM treatment process for rail applications.

## 4. Conclusions

The surface layer of the normal and heat-treated rails was hardened by the UNSM treatment process. The compressive residual stress was also induced in the surface layer by severe plastic deformation generated by the UNSM treatment process. The heat treatment of the rail was good enough to improve the wear resistance, but the effect of the UNSM treatment process was revealed to be greater than that of heat treatment on the wear resistance. The wear amount of the heat-treated rail was found to be greater than that of the UNSM-treated rail with increasing the rotating speed. The RCF lifetime of the rail was extended after the UNSM treatment process. The fatigue lifetime of the rail specimens was reduced with increasing the contact pressure, but the ratio was reduced by the UNSM treatment process. The surface damage and fatigue lifetime of the wheel-rail interaction play a fundamental role in determining the reliability and performance of the whole wheel-rail system. Hence, it is expected, according to the obtained results, that the wear and fatigue of the wheel-rail interaction may be improved with the application of the UNSM treatment process since the harder material provides a benefit in reducing whole-system maintenance and repairing costs. Dynamic tests, which can simulate a similar condition to the real wheel-rail interface, need to be performed in order to apply this treatment to a real field.

## Figures and Tables

**Table 1:** The chemical composition of 60 kgK rail steels in (wt %).

Rails	C	Si	Mn	P	S	Fe
Normal	0.72	0.34	0.80	0.025	0.025	Balance
Heat-treated	0.80	0.35	1.00	0.030	0.020	

**Table 2:** Optimized UNSM treatment parameters for 60 kgK rail steel.

Frequency, kHz	Amplitude, µm	Speed, mm/min	Impact Load, N	Feed-Rate, mm	Ball Diameter, mm	Ball Material
20	30	3000	40	0.07	2.38	WC

**Table 3:** RCW test conditions.

Load, N	Rolling Speed, rpm	Rolling Time, h	Condition
50	500	1	dry
	1000		
	1500		

**Table 4:** RCF test conditions.

Contact Stress, GPa	Rolling Time, h	Rolling Speed, rpm	Condition
2.5	12, 24	1000	dry
3.0			

**Table 5:** Comparison of cycles to failure of the normal and UNSM-treated normal rail specimens obtained by RCF tests with respect to contact stress.

Contact Stress, GPa	Cycles to Failure	
	Normal	Normal UNSM-Treated
2.5	478,000	669,000
3.0	416,000	651,000
